# Clinical Lectures

**Published:** 1873-04-15

**Authors:** 


CLINICAL LECTURES.—The volume of Clinical Lectures, which was announced for the middle of March, was delayed a little by the publisher, but is now ready for sale. Copies have been mailed to all who had sent orders for it; and if any more of our readers prefer to send directly to the office of THE EXAMINER for the work, they will be promptly supplied by mail, postage paid.

